# Manipulation of β‐carotene levels in tomato fruits results in increased ABA content and extended shelf life

**DOI:** 10.1111/pbi.13283

**Published:** 2019-12-24

**Authors:** Gianfranco Diretto, Sarah Frusciante, Claudia Fabbri, Nicolas Schauer, Lucas Busta, Zhonghua Wang, Antonio J. Matas, Alessia Fiore, Jocelyn K.C. Rose, Alisdair R. Fernie, Reinhard Jetter, Benedetta Mattei, Jim Giovannoni, Giovanni Giuliano

**Affiliations:** ^1^ Italian national Agency for New technologies, Energy, and Sustainable Development (ENEA) Casaccia Research Center Roma Italy; ^2^ Department of Biology and Biotechnology Sapienza University of Rome Rome Italy; ^3^ Max‐Planck‐Institut für Molekulare Pflanzenphysiologie Potsdam‐Golm Germany; ^4^ Department of Chemistry University of British Columbia Vancouver BC Canada; ^5^ Center for Plant Science Innovation and Department of Biochemistry University of Nebraska–Lincoln Lincoln NE USA; ^6^ Department of Botany University of British Columbia Vancouver BC Canada; ^7^ College of Agronomy Northwest A&F University Yangling China; ^8^ Plant Biology Section School of Integrative Plant Science Cornell University Ithaca NY USA; ^9^ Department of Plant Biology Institute for Mediterranean and Subtropical Horticulture “La Mayora” (IHSM‐UMA‐CSIC) University of Málaga Málaga Spain; ^10^ Department of Health, Life and Environmental Sciences University of L'Aquila L'Aquila Italy; ^11^ U.S. Department of Agriculture–Agricultural Research Service Robert W. Holley Center for Agriculture and Health Ithaca NY USA; ^12^ Boyce Thompson Institute for Plant Research Cornell University Ithaca NY USA

**Keywords:** tomato, β‐carotene, ABA, ripening

## Abstract

Tomato fruit ripening is controlled by the hormone ethylene and by a group of transcription factors, acting upstream of ethylene. During ripening, the linear carotene lycopene accumulates at the expense of cyclic carotenoids. Fruit‐specific overexpression of *LYCOPENE* β*‐CYCLASE* (*LCYb*) resulted in increased β‐carotene (provitamin A) content. Unexpectedly, *LCYb*‐overexpressing fruits also exhibited a diverse array of ripening phenotypes, including delayed softening and extended shelf life. These phenotypes were accompanied, at the biochemical level, by an increase in abscisic acid (ABA) content, decreased ethylene production, increased density of cell wall material containing linear pectins with a low degree of methylation, and a thicker cuticle with a higher content of cutin monomers and triterpenoids. The levels of several primary metabolites and phenylpropanoid compounds were also altered in the transgenic fruits, which could be attributed to delayed fruit ripening and/or to ABA. Network correlation analysis and pharmacological experiments with the ABA biosynthesis inhibitor, abamine, indicated that altered ABA levels were a direct effect of the increased β‐carotene content and were in turn responsible for the extended shelf life phenotype. Thus, manipulation of β‐carotene levels results in an improvement not only of the nutritional value of tomato fruits, but also of their shelf life.

## Introduction

Plants have evolved several mechanisms for seed dispersal, one of which is the development of fleshy fruits with attractive organoleptic characteristics, such as fleshiness, colours and flavours able to attract frugivore animals for seed dispersal. Tomato (*Solanum lycopersicum* L.) is a model system for the study of fruit ripening, mainly due to many genetic and postgenomic resources available for this species (reviewed in refs. Gascuel *et al.*, 2017; Giovannoni *et al.*, 2017; Klee and Giovannoni, 2011; Seymour *et al.*, 2013). Tomato fruit development comprises an initial phase of postanthesis cell division, followed by one of cell expansion, during which concentrations of the hormones ethylene and abscisic acid (ABA) are both low (Zhang *et al.*, 2009). Immediately, after the mature green (MG) stage of ripening, a transient peak in ABA content occurs, followed by a switch of ethylene production from System 1 (autoinhibitory) to System 2 (autocatalytic) and a peak in ethylene production (Klee and Giovannoni, 2011; Seymour *et al.*, 2013). Several other events follow, such as fruit softening, the accumulation of sugars and organic acids, and a change of colour from green to red, due to the accumulation of the linear carotene, lycopene (Giuliano *et al.*, 1993; Klee and Giovannoni, 2011). These changes are accompanied, at the molecular level, by extensive changes in gene expression (Alba *et al.*, 2005; Carbone *et al.*, 2005), with lycopene accumulation being highly associated with the up‐regulation of genes encoding *PHYTOENE SYNTHASE 1* (*PSY1*) and *PHYTOENE DESATURASE* (*PDS*) (Giuliano *et al.*, 1993), and the down‐regulation of genes encoding *LYCOPENE* β*‐* and ε*‐CYCLASE* (*LCYb* and *LCYe*) (Pecker *et al.*, 1996; Ronen *et al.*, 1999; Ronen *et al.*, 2000). Many of these events have been demonstrated to depend on the presence of a functional ethylene receptor (Alba *et al.*, 2005).

**Figure 1:**
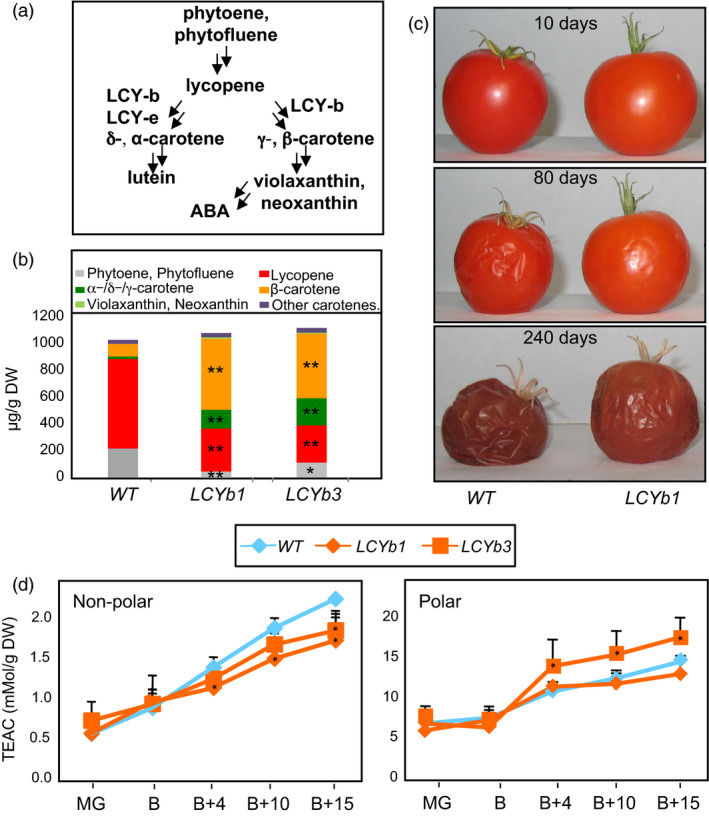
Carotenoid composition, shelf life and antioxidant activity of *LCYb*‐overexpressing fruits. (a) Schematic representation of β‐carotene/ABA biosynthesis. (b) Carotenoid content of *WT* and *LCYb*‐overexpressing fruits at B+10. Minimum levels of detection were 0.01 mg/g DW. Data are the average ± stdev of three biological replicates. (c) Elongated shelf life of *LCYb*‐overexpressing fruits. Fruits were harvested at the B stage and kept at 20  2 °C, 60  5% relative humidity for the indicated periods of time. (d) Antioxidant activity, measured at different ripening stages as Trolox equivalent antioxidant capacity (TEAC) in nonpolar (left) and polar (right) extracts. MG: mature green; B: breaker; B+4, B+10, B+15: days after breaker stage. Data are the average ± stdev of three biological replicates. Asterisks indicate significant differences from *WT* according to a Student's *t*‐test (*P < 0.05, **P < 0.01).

Besides ethylene, tomato fruit ripening is controlled by a cascade of transcription factors, some of which mediate input by other hormones, such as auxin and ABA (Giovannoni *et al.*, 2017; Klee and Giovannoni, 2011; Seymour *et al.*, 2013). Exogenous application of ABA at the mature green (MG) stage increases the amplitude of the ethylene peak and accelerates ripening, while the application of fluridone (a carotenoid biosynthesis inhibitor) or nordihydroguaiaretic acid (an inhibitor of ABA biosynthesis) has the opposite effect (Zhang *et al.*, 2009). Knockout mutations of *ZEAXANTHIN EPOXIDASE *(*ZEP*) or silencing of *9‐cis‐EPOXYCAROTENOID DIOXYGENASE* (*NCED*) results in decreased endogenous ABA and increased ethylene (Galpaz *et al.*, 2008; Ji *et al.*, 2014), respectively. In contrast, silencing of three *CYP707A2* isoforms, encoding *ABA 8′‐hydroxylases* acting in ABA catabolism, or of *ABA uridine diphosphate glucosyltransferase* (*SlUGT75C1*), which produces esterified ABA‐glucose, generated over‐ripe fruits with increased ABA levels (Ji *et al.*, 2014; Sun *et al.*, 2017). A similar phenotype was observed in RNAi lines for *SlPP2C1*, a *group A type 2C protein phosphatase* involved in ABA signalling (Zhang *et al.*, 2018). These observations suggest that ABA influences fruit ripening in different ways, depending on the mode (external or endogenous) and timing of the application. Since ABA is synthesized from β‐xanthophylls (Figure 1A), an interesting corollary of the above hypothesis is that carotenoid composition may itself play a role in controlling tomato fruit ripening. At the MG stage, levels of β‐xanthophylls are high, and during ripening, they decline, due to the down‐regulation of *LCYb* genes (Pecker *et al.*, 1996; Ronen *et al.*, 2000), possibly affecting ABA levels.

Several reports have described the metabolic engineering of plant carotenoid contents (Giuliano, 2014; Giuliano, 2017). Overexpression of a *LCYb* gene from Arabidopsis under the control of the ripening‐associated *PDS* promoter leads to ripe tomato fruits that accumulate high levels of β‐carotene (Rosati *et al.*, 2000). Apart from the transgene, these engineered lines are perfectly isogenic with their lycopene‐accumulating parental genotype, making them a good system to study the possible influence of carotenoid composition on fruit ripening. Using two independent transgenic lines, we conducted a system‐wide study of the effect of increased β‐carotene levels on tomato fruit ripening and shelf life. Our data suggest that the increase in the β‐carotene content results in higher ABA content, which in turn has an effect on fruit ripening and shelf life.

## Results

### *LCYb*‐overexpressing fruits exhibit higher β‐carotene levels and increased shelf life, which does not correlate with antioxidant activity

Transgenic tomato lines overexpressing Arabidopsis *LCYb* under the control of the chromoplast‐associated *PDS* promoter accumulate β‐carotene in ripe fruits (Rosati *et al.*, 2000; Figure 1B,C). Homozygous T4 lines derived from two independent transformation events were grown in the greenhouse, and fruits were harvested at five stages of ripening: mature green (MG), breaker (B), breaker+4 or pink (B+4), breaker+10 or ripe (B+10) and breaker+10 or over‐ripe (B+15 and B+40). Expression of *AtLCYb* peaked at B and B+4 (Figure S1). A fourfold to 10‐fold increase in β‐carotene and other cyclic carotenes and a twofold to threefold decrease in linear carotenes were observed in B+10 fruits compared to *WT*. Xanthophylls were undetectable in *WT* fruits, but became detectable in *LCYb*‐overexpressing fruits (Figure 1C, Table S1).

*LCYb*‐overexpressing fruits stored at room temperature for several months exhibited, relative to *WT,* a significant increment in shelf life, increased firmness and reduced water loss (Figure 1D). No significant alteration in the time elapsing between anthesis and fruit breaker stage was observed (Figure S2), indicating that the alteration was confined to the late stages of fruit development. Since the observed changes in carotenoid levels in *LCYb*‐overexpressing fruits are likely to affect fruit antioxidant activity, which is known to impact fruit shelf life (Zhang *et al.*, 2013), we measured the antioxidant activity in both polar and nonpolar extracts of *WT* and *LCYb*‐overexpressing fruits at five ripening stages (MG, B, B+4, B+10 and B+15; Figure 1D). No significant differences compared to the *WT* were observed at early stages (MG and B). At later stages (B+4 through B+15), *LCYb*‐overexpressing fruits displayed a reduction in the antioxidant activity of the nonpolar fraction, while that of the polar fraction varied in opposite directions in the two transgenic lines, with the *LCYb3* line showing an increase and the *LCYb1* line a decrease with respect to the *WT*.

### Increased firmness and decreased water loss of *LCYb*‐overexpressing fruits correlate with altered cell wall and cuticle composition

**Figure 2:**
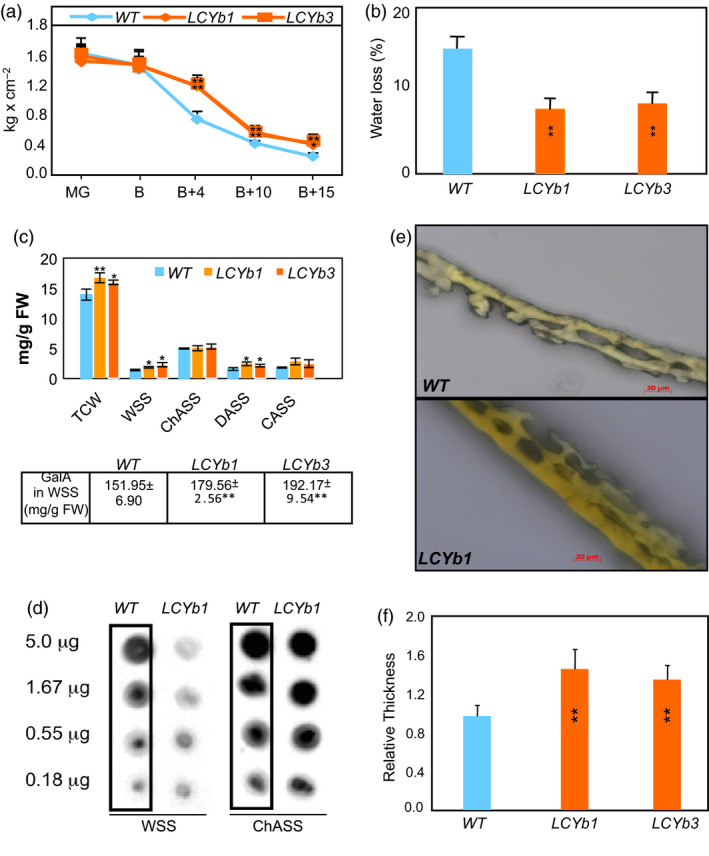
Altered firmness, water loss, cuticle thickness, cell wall and cuticle composition of *LCYb*‐overexpressing fruits. (a) Flesh firmness at five ripening stages, measured with a hand‐held penetrometer. Data are the average ± stdev of 10 biological replicates. (b) Water loss between the MG and B+15 ripening stages. Data are the average ± stdev of 10 biological replicates. (c) Amounts of the different cell wall fractions in *WT* and *LCYb‐*overexpressing fruits at B+10. TCW: total cell walls; WSS: water‐soluble solids; CHASS: chelating agent‐soluble solids; DASS: dilute alkali‐soluble solids; CASS: concentrated alkali‐soluble solids. Data are the average ± stdev of at least three biological replicates. (d) Methyl‐esterified pectin content of cell walls at B+10. Identical amounts of carbohydrate were applied to nitrocellulose membranes and probed with the LM20 monoclonal antibody. (e) Light microscopy of cuticles from *WT* and *LCYb‐*overexpressing fruits at B+10. (f) Cuticle thickness of *WT* and *LCYb‐*overexpressing fruits at B+10. Data are the average ± stdev of 10 biological replicates. Significant differences from *WT* were evaluated using a Student's *t*‐test (*P < 0.05, **P < 0.01).

The firmness of *WT* and *LCYb*‐overexpressing fruits was measured with a hand‐held penetrometer at five different ripening stages. At MG and B, the firmness of the two types of fruits was similar, and then starting at B+4 and until the end of ripening, *LCYb*‐overexpressing fruits exhibited significantly increased firmness (Figure 2A). One of the components of fruit firmness, evapotranspiration, was found to be significantly lower in *LCYb*‐overexpressing fruits (Figure 2B). Total cell wall extracts from the pericarp of B+10 fruits were fractionated by sequential extraction with water, chelating agent, dilute alkali and concentrated alkali (Huisman *et al.*, 1998). Significant increases were observed in the abundance of total cell wall material, water‐soluble solids and dilute alkali‐soluble solid fractions of *LCYb*‐overexpressing fruits. Additionally, the WSS fraction of *LCYb*‐overexpressing fruits showed a significant increase in the abundance of galacturonic acid, which is present in the backbone of HG and RG‐I polysaccharides (Figure 2C). The occurrence of methyl‐esterified pectins in the WSS fraction was analysed by immunodot analysis using the LM20 monoclonal antibody, which recognizes highly methyl‐esterified HG epitopes. A lower abundance of methyl‐esterified pectins was observed in the WSS fraction from *LCYb‐*overexpressing fruits, compared to *WT,* while the ChASS fraction did not show significant differences (Figure 2D).

Cuticle thickness was also significantly increased in *LCYb*‐overexpressing B+10 fruits (Figure 2E,F). We further investigated the chemical composition of the cutin polymer and the associated cuticular waxes using gas chromatography–mass spectrometry (GC‐MS). The amounts of all cutin monomers were significantly higher in the *LCYb‐*overexpressing fruits than in *WT*. Among the cuticular waxes, the major very‐long‐chain acyl derivatives were relatively unchanged in *LCYb*‐overexpressing fruits, while the triterpenoid components α‐amyrin, taraxasterol, ψ‐taraxasterol and δ‐amyrin were all >2‐fold greater than *WT* levels (Table S2).

### Metabolic remodelling in *LCYb‐*overexpressing fruits

A total of 72 phenylpropanoids were measured in both the flesh and the cuticle of B+10 fruits, using liquid chromatography coupled with high‐resolution mass spectrometry (LC‐HRMS; Table S3). Most compounds showed an overaccumulation in *LCYb vs WT* fruit cuticles. A notable exception was found in the group of phenolic compounds, such as 1‐caffeoyl‐1‐beta‐D‐glucose, 4‐*p*‐coumaroylquinic acid, chlorogenic, coumaric, dicaffeoylquinic acid and ferulic acids, which showed a slight decrease in the flesh of *LCYb*‐overexpressing fruits relative to *WT*; on the contrary, stronger positive variations were observed for flavonoids and flavonoid glycosides: 26 out of 48 metabolites showed significantly higher levels in *LCYb*‐overexpressing fruits, with catechin/epicatechin, eriodictyol, kaempferol and quercetin conjugates showing the largest increases (Table S3).

Additionally, the levels of 58 metabolites (20 amino acids, 19 sugars/polyols, eight organic acids and 11 others) were quantified by GC‐MS in ripe fruits of two *LCYb*‐overexpressing lines (Schauer *et al.*, 2005; Table S4). Thirteen compounds (five amino acids, seven sugars/polyols and putrescine) showed significant changes in both transgenic lines. The amino acids alanine, aspartate, phenylalanine and proline, the sugars mannitol and sucrose, the organic acid 2‐oxo‐butyric acid and the polyamine putrescine were lower in *LCYb‐*overexpressing fruits. In contrast, the sugars erythritol, galactinol, glucose, glucoheptose and melibiose, and the sugar phosphate fructose 6‐P were higher. When averaged between the two *LCYb* lines, all changes were less than twofold in magnitude (Table S4), suggesting that changes in primary metabolism were minor.

### *LCYb*‐overexpressing fruits exhibit increased ABA and decreased ethylene production and altered expression of the *RIN* and *NOR* ripening regulators

**Figure 3:**
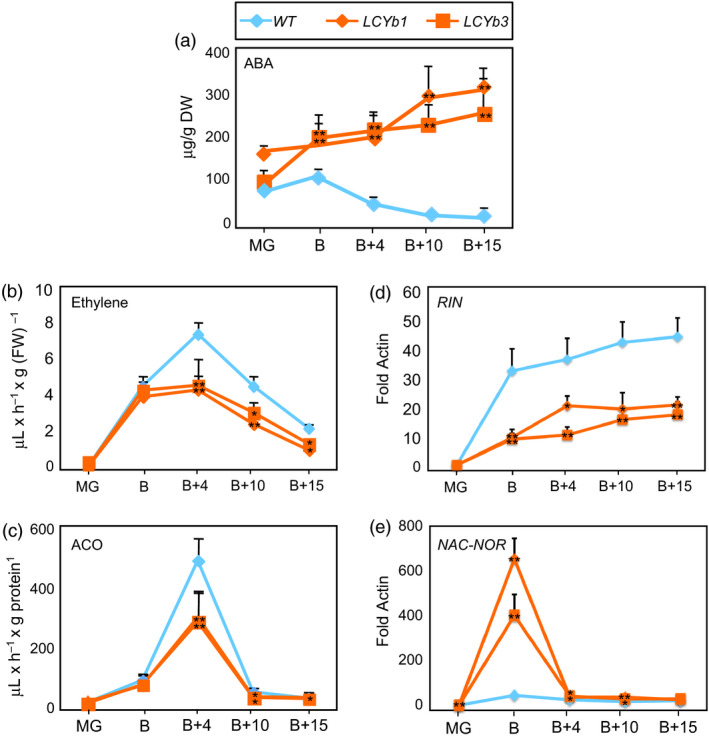
ABA/ethylene metabolism and *RIN/NOR* expression during fruit ripening. (a) ABA content of *WT* and *LCYb‐*overexpressing fruits at different stages of ripening. (b) Ethylene production in *WT* and *LCYb‐*overexpressing fruits at different stages of ripening. (c) ACO activity in extracts from *WT* and *LCYb‐*overexpressing fruits at different stages of ripening. (d, e) *RIN* and *NAC‐NOR* expression in *WT* and *LCYb‐*overexpressing fruits*,* measured by qRT‐PCR. Data are normalized on the expression level of the actin housekeeping gene. Data are the average ± stdev of 3 (a–e) or 10 (b) biological replicates. Asterisks indicate statistical significance (*0.05 < *P*; **0.01 < *P*) in a Student's *t*‐test.

ABA is synthesized from violaxanthin and neoxanthin, which are increased in *LCYb*‐overexpressing fruits (Figure 1A,B). ABA accumulation during ripening was measured using LC‐HRMS (Figure 3A, Table S5) and showed very distinct kinetics in *WT* and *LCYb*‐overexpressing fruits: in *WT* fruits, ABA levels peaked at the B stage and then progressively declined until B+15, while in *LCYb‐*overexpressing fruits they showed a much larger peak at the B stage, followed by a further increase to 17‐fold *WT* levels at B+15. ABA catabolites (phaseic acid, dihydrophaseic acid, ABA‐Glc and 7‐hydroxy‐ABA) also showed increased levels in *LCYb‐*overexpressing fruits (Table S5). These data indicate that *LCYb* overexpression during ripening causes accumulation of β‐carotene and β‐xanthophylls, and that the increased flux through the β‐branch of carotene biosynthesis results in increased levels of ABA and its downstream catabolites.

We also measured ethylene emission by intact fruits and the enzymatic activity of ACC oxidase, the last enzyme in the ethylene biosynthetic pathway. Ethylene production and ACC oxidase activity both peaked at B+4 in *WT* fruits, and the peak showed an approximately 50% reduction in *LCYb*‐overexpressing fruits relative to *WT* (Figure 3B,C).

The kinetics of expression of two key regulators of fruit ripening, *RIN* (Vrebalov *et al.*, 2002) and *NAC‐NOR* (Giovannoni *et al.*, 2004; Osorio *et al.*, 2011), were analysed by quantitative real‐time RT‐PCR (qRT‐PCR) in *WT* and *LCYb*‐overexpressing fruits (Figure 3D,E). The *RIN* transcript was strongly repressed starting at the B stage and throughout the whole ripening process, while, on the contrary, *NAC‐NOR* displayed a large increase in expression at the B stage.

### Systems analysis of *WT* and *LCYb*‐overexpressing fruits

Transcript profiling was performed on fruits at three ripening stages (MG, B and B+10) in the *WT* and two *LCYb*‐overexpressing lines using the EU‐TOM3 Affymetrix microarray (Tables S6, S8 and S10A,B). Genes up‐ or down‐regulated >1.5‐fold in both transgenic lines with respect to *WT*, with a *P*‐value ≤0.05, were considered differentially regulated and are shown as MapMan representations in Figure S3. A total of 123 transcripts were found to be differentially regulated in both the MG and B stages, 137 in both B and B+10 and 148 in both MG and B+10 (Figure S4, Table S12). GO enrichment analysis (Tables S7, S9 and S11A,B) showed a series of enriched GO terms in up‐regulated genes (tetrapyrrole/chlorophyll and protein binding and amine metabolism at the MG and B+10 stages, respectively) and in down‐regulated ones (carbohydrate and nucleotide metabolism). We also performed a manual annotation of differentially regulated transcripts involved in well‐known aspects of fruit ripening, including ethylene metabolism and regulation, cell wall remodelling, cuticle biogenesis, primary metabolism, phenylpropanoid, carotenoid and apocarotenoid pathways (including ABA). All of the aforementioned classes were represented in differentially regulated genes, with ethylene‐ and cell wall‐related genes showing the highest number of differentially expressed (particularly down‐regulated) representatives (Tables S6, S8 and S10A,B). Interestingly, a series of key genes in the phenylpropanoid pathway (*PHENYLALANINE AMMONIA‐LYASE* (*PAL*) and *CHALCONE SYNTHASE* (*CHS*) at the MG stage; *PAL* and *4‐COUMARATE:COA LIGASE 2* (*4CL2*) at the B stage; *CHS* and *DIHYDROFLAVONOL 4‐REDUCTASE* (*DFR*) at the B+10 stage) were also overexpressed in *LCYb*‐overexpressing fruits compared to the *WT*.

The levels of 28 additional transcripts involved in fruit ripening control were measured in B+10 fruits using through qRT‐PCR (Table S13). The majority of transcription factors, including *RIN*, *TAGL1* (Itkin *et al.*, 2009; Vrebalov *et al.*, 2009), *CNR* (Manning *et al.*, 2006), *HB‐1* (Lin *et al.*, 2008), *AP2a* (Chung *et al.*, 2010; Karlova *et al.*, 2011) and *TDR4/FUL1* (Bemer *et al.*, 2012), were down‐regulated in *LCYb‐*overexpressing fruits. Down‐regulated transcripts also included the *NEVER‐RIPE* ethylene receptor, *GR*, *CTR1* and *EIN2,* participating in ethylene signalling (Barry *et al.*, 1996; Fu *et al.*, 2005), and *ACS2, ACS4* and *ACO1* genes (Barry *et al.*, 2000; Bidonde *et al.*, 1998) involved in ethylene biosynthesis. Notable exceptions to this pattern were the *NAC‐NOR* transcription factor (Giovannoni *et al.*, 2004; Osorio *et al.*, 2011), and the *E4* and *E8* ethylene‐inducible genes (Cordes *et al.*, 1989), which were up‐regulated in *LCYb‐*overexpressing fruits. Several genes involved in cell wall degradation/remodelling were also down‐regulated, such as *POLYGALACTURONASE 1 *(*PG1*)*, PECTATE LYASE *(*PL*)*,* α*‐L‐ARABINOFURANOSIDASE 1 *(*ARF1*)*, EXPANSIN 1 *(*EXP1*)*,* β‐*D‐XYLOSIDASE 1* and* 2 *(*XYL1, XYL2*) and *XYLOGLUCAN ENDO‐TRANSGLUCOSYLASE/HYDROLASE 4* and *8 *(*SIXTH4, SIXTH8*) genes. Exceptions included the *PECTIN METHYLESTERASE 1* and *2* (*PMEU1, PMEU2*) genes, which were up‐regulated, consistent with the decreased levels of pectin methyl‐esterification noted above.

**Figure 4:**
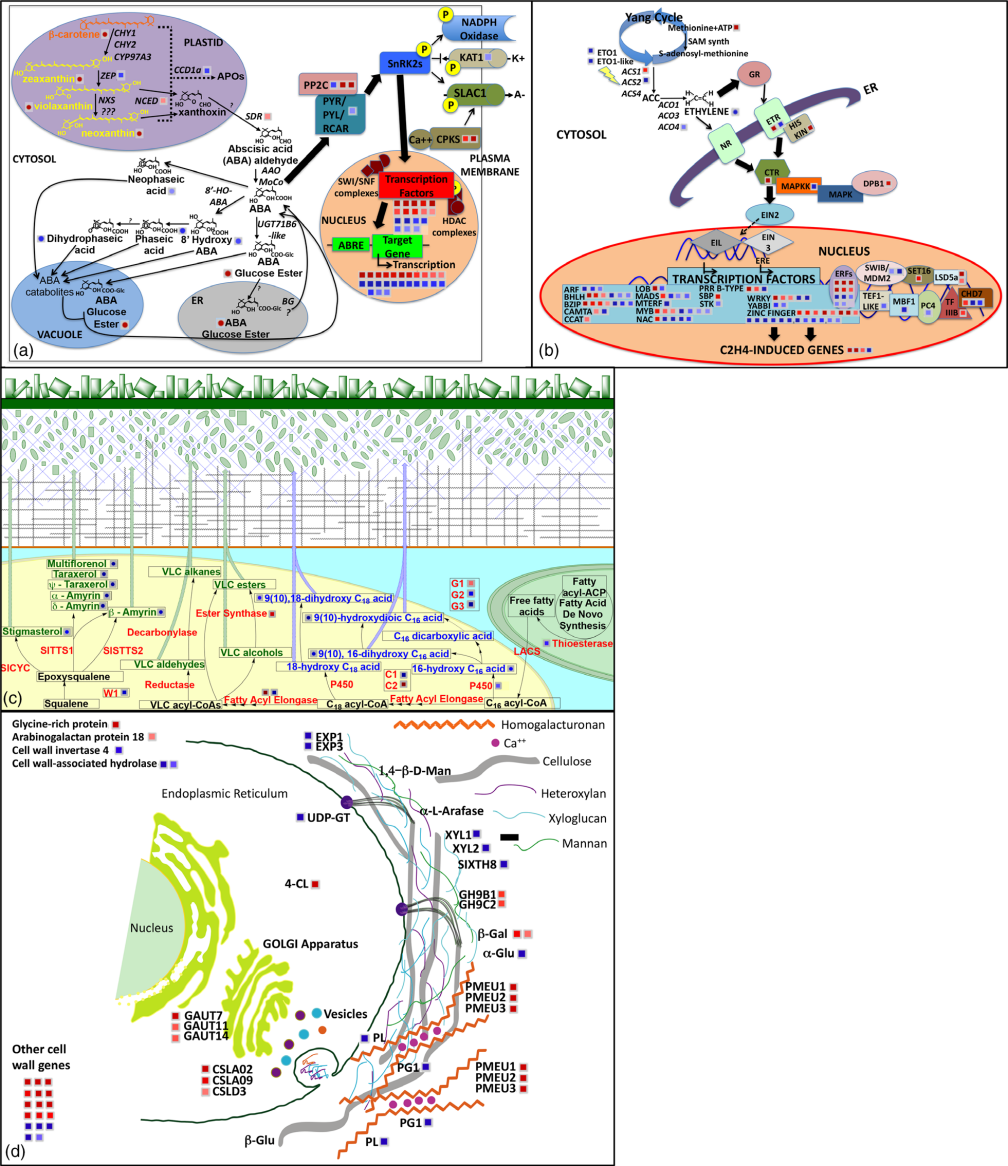
MapMan representation of transcriptional‐metabolic perturbations in *LCYb‐*overexpressing fruits at B+10. Genes are represented by squares and metabolites by circles. Red indicates induction, and blue indicates repression in the *LCYb1* and *LCYb3* lines with respect to the *WT*. (a) ABA metabolism/signal transduction (54 Genes, 10 metabolites); (b) Ethylene metabolism/signal transduction (119 Genes, 1 metabolite). (c) Cuticle biogenesis (11 Genes, 13 metabolites); (d) Cell wall biogenesis (49 Genes, 1 metabolite).

The metabolic and transcriptional alterations observed in *LCYb*‐overexpressing fruits at B+10, associated with ABA, ethylene, cuticle and cell wall metabolism, are summarized as *ad hoc* MapMan charts (Thimm *et al.*, 2004) in Figure 4. *LCYb* overexpression in fruits resulted in extensive perturbations of ABA and ethylene metabolism and signal transduction, and also of cuticle and cell wall biogenesis.

**Figure 5:**
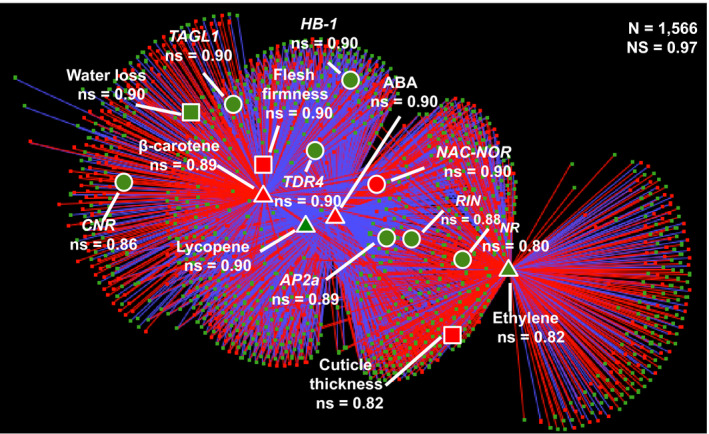
Network analysis of co‐regulated traits in *LCYb‐*overexpressing fruits at B+10. Correlation network of 1566 traits differentially regulated in *LCYb‐*overexpressing fruits at B+10 with respect to the corresponding WT stage. Specific nodes of interest, associated with fruit ripening, are highlighted, together with their node strengths (ns, Diretto et al, 2010a). N, number of nodes. NS, network strength (Diretto et al, 2010a). Each node represents a transcript (circle), a metabolite (diamond) or a phenotypic trait (square). For more details, see ‘Methods’.

In order to identify co‐regulated traits (e.g. metabolites, hormones, transcripts, phenotypic traits), we chose 1612 variables that are differentially regulated in *LCYb‐*overexpressing with respect to *WT* fruits. The Pearson correlation coefficient values (ρ) for the resulting trait pairs (Table S14) were used to build a correlation network (Diretto *et al.*, 2010a), including correlations |ρ| > 0.90 (Figure 5, Table S15). The overall ‘network strength’ (i.e. the average of all |ρ| values (Diretto *et al.*, 2010a) was very high (0.97), indicating that the variables are tightly co‐regulated. Several metabolites, hormones and phenotypic traits associated with fruit ripening grouped as a tight cluster, in a region populated by known ripening regulators. Notably, ABA had a central position in this network and was strongly co‐regulated with *NAC*‐*NOR* (ρ = 0.99), *RIN* (ρ = −0.99) and *AP2a* (ρ = −0.99)*,* and less so with ethylene, *NR, HB‐1*, *CNR* and *TAGL1*. *NAC*‐*NOR, RIN* and *AP2a* were also strongly co‐regulated with ethylene (ρ = 0.96, 0.98 and 0.97, respectively). Interestingly*,* the *CNR* ripening regulator (Manning *et al.*, 2006) was more strongly co‐regulated with ABA (−0.90) than with ethylene (0.73). We also constructed subnetworks centred around ABA and ethylene. The ABA network (Figure S5A, Table S16A) included the vast majority of transcription factor genes known to control fruit ripening: *NAC‐NOR*, *TDR4*, *AP2A*, *HB‐1*, *RIN*, *TAGL1* and *CNR*. Of these, *NAC‐NOR* showed positive co‐regulation with ABA, while all other genes showed negative co‐regulation (Tables S15 and S16A). Additional genes strongly co‐regulated with ABA included genes involved in ABA signal transduction; genes for ethylene biosynthesis, sensing and signal transduction; and genes for carotenoid, chlorophyll and cell wall metabolism (Table S16A). In the second network, centred around ethylene (Figure S5B; Table S16B), strongly co‐regulated genes were involved in ethylene biosynthesis, sensing and signal transduction, but also key ripening regulators and genes involved in ABA signal transduction (Tables S15 and S16B). This is consistent with recent suggestions of substantial crosstalk between the networks controlling these hormones during ripening (Galpaz *et al.*, 2008; Ji *et al.*, 2014; Sun *et al.*, 2017).

### Abamine treatment of *LCYb*‐overexpressing fruits reduces ABA accumulation, increases ethylene production and reverses the long shelf life phenotype

**Figure 6:**
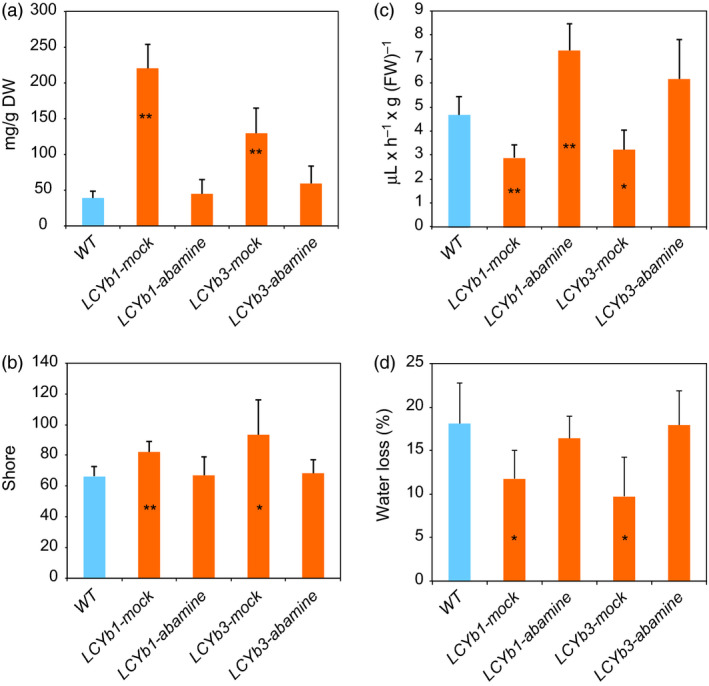
Abamine treatment reverses the phenotype of *LCYb*‐overexpressing fruits. (a) ABA content in *WT* and *LCYb‐*overexpressing fruits at B+10, mock‐treated or treated with 1 mm abamine; (b) Flesh firmness of *WT* and *LCYb‐*overexpressing fruits at B+10, mock‐treated or treated with 1 mm abamine; (c) Ethylene emission of *WT* and *LCYb‐*overexpressing fruits at B+10, mock‐treated or treated with 1 mm abamine; (d) Water loss of *WT* and *LCYb‐*overexpressing fruits, mock‐treated or treated with 1 mm abamine, between MG and B+10. Data are the average ± stdev of five biological replicates. Asterisks indicate significant differences from *WT* according to Student's *t*‐test (*P < 0.05, **P < 0.01).

Abamine is a well‐known inhibitor of NCED enzymatic action and of ABA biosynthesis (Han *et al.*, 2004). To better investigate the ABA role in the extended shelf life phenotype, we treated *LCYb*‐overexpressing fruits with abamine at the MG stage. The treatment resulted in a reduction of ABA in *LCYb*‐overexpressing fruits to levels similar to *WT* ones (Figure 6A). As a result, ethylene production was increased to levels slightly higher than those of *WT* fruits (probably as a result of the injection), while flesh firmness and water loss reverted to *WT* levels (Figure 6B–D).

## Discussion

*LCYb* overexpression in tomato fruits resulted in increased β‐carotene content and fruit firmness and in extended shelf life. At the biochemical level, this phenotype was accompanied by a modification of cell wall composition and polymerization, of cuticle thickness and chemical composition, of primary metabolite and phenylpropanoid profiles in the fruit pericarp. This is, to our knowledge, the first time that metabolic engineering of carotenoid biosynthesis has been reported to have such profound and pleiotropic effects on fruit ripening. Until now, carotenoid composition has merely been considered as an output of the regulatory circuit controlling fruit ripening.

### Metabolic alterations in *LCYb*‐overexpressing fruits

Some of the metabolic perturbations observed in *LCYb*‐overexpressing fruits can be attributed to their delayed ripening: compounds such as alanine, aspartate, proline, mannitol and putrescine increase during normal fruit ripening (Carrari *et al.*, 2006), and show a decrease in ripe *LCYb*‐overexpressing fruits compared to *WT* ones. Other metabolic perturbations can be attributed to the observed changes in gene expression: for instance, the decrease in sucrose and increase in glucose levels correspond to an induction, at the B stage, of *ACID INVERTASE*, which hydrolyses sucrose into glucose and fructose. The functional role of acid invertase is very well characterized in tomato fruits, with quantitative trait loci, genome‐wide association studies and reverse genetic approaches, all indicating its importance for determining soluble solid content (Fridman *et al.*, 2004; Tieman *et al.*, 2017) and aspects of fruit development and seed yield (Zanor *et al.*, 2009). Also, overexpression of *ACID INVERTASE* has been observed in tomato fruits overexpressing an ABA response element binding factor (*SlAREB1*) which leaves ethylene levels unaltered (Bastias *et al.*, 2011), indicating that this trait may be under direct ABA control. Interestingly, *LCYb‐*overexpressing fruits share considerable commonalities with those of the well‐characterized ripening mutants *rin, nor, NR* and *ap2* (Karlova *et al.*, 2011; Osorio *et al.*, 2011) with decreases in alanine, aspartic acid, glutamic acid, glutamine, phenylalanine and proline all being observed in *rin, nor* and *NR* and the changes in aspartic acid, glutamine, phenylalanine and proline also being observed in *ap2.* Glucose also showed consistent trends between ripening mutants and *LCYb*‐overexpressing fruits, being overaccumulated in all the genotypes; while sucrose, present at higher contents in *nor*, *rin* and *NR*, displayed an opposite trend, that is reduction, in *LCYb*‐overexpressing fruits.

Additional metabolic fluctuations that can be attributed to ABA accumulation are the increase in flavonoids. The extent of changes (>10‐fold for some flavonoid glycosides) suggests they represent direct effects of the genetic manipulation and thus of the increase in ABA in *LCYb*‐overexpressing fruits, compared to the less pronounced alterations observed in primary metabolites, which are likely to represent secondary effects. This hypothesis finds support in a series of previous studies linking ABA and flavonoid levels in apple (Lu *et al.*, 2017), soybean (Gupta *et al.*, 2018) and tomato (Mou *et al.*, 2015). In agreement with biochemical data, a series of key structural phenylpropanoid genes was up‐regulated in *LCYb‐*overexpressing fruits: for instance, *PHENYLALANINE AMMONIA‐LYASE* (*PAL*) at the MG and B stages, and *CHALCONE SYNTHASE* (*CHS*) at the MG and B+10 stages. ABA has been shown previously to promote *PAL* and *CHS* expression (Yu *et al.*, 2015; Zhang *et al.*, 2017) and PAL activity (Jiang and Joyce, 2003).

Purple tomatoes, overexpressing *Del* and *Ros* transcription factors from snapdragon and accumulating large amounts of anthocyanins, display extended shelf life, a phenotype attributed to the increased total antioxidant activity caused by anthocyanin accumulation (Zhang *et al.*, 2013). While we cannot exclude that the increase of flavonoid levels in the peel of *LCYb*‐overexpressing fruits influences its permeability, and hence, water loss and total antioxidant activity do not seem to have a causal relationship with the extended shelf life of these fruits: the fluctuations of total antioxidant activity in the polar fraction did not correlate with fruit shelf life, while those in the nonpolar fraction showed a negative correlation. This finding is not surprising, since β‐carotene has been reported to have a lower antioxidant activity compared to lycopene (Bohm *et al.*, 2002). Thus, the conversion of large part of lycopene into β‐carotene in *LCYb*‐overexpressing fruits is consistent with the observed decrease of antioxidant activity in the nonpolar fraction.

### Alterations in cell wall and cuticle composition

The extraction and subsequent fractionation of the cell walls of *LCYb‐*overexpressing fruits yielded higher amounts of total cell walls, water‐soluble and diluted alkali‐soluble solids per unit weight compared to *WT*, indicating alterations both in the content and in the solubility of cell wall polymers. The higher content of the pectic backbone sugar GalA in the WSS fraction suggested a higher content of pectins, which were less methyl‐esterified than those from *WT* fruits. The regulated swelling and disassembly of primary cell walls and the modification of the middle lamellae between adherent primary cell walls are thought to be important factors contributing to tissue softening during tomato fruit ripening. The increased abundance in *LCYb‐*overexpressing fruits of linear, low‐esterified homogalacturonan is likely to influence both cell adhesion and fruit texture. Pectins secreted to the cell wall possess methyl‐ester side chains, which are removed by pectin methylesterase (PME) as a prerequisite for polygalacturonase (PG) action (Wakabayashi *et al.*, 2003). PME can play dual and contrasting roles within the plant cell wall: on the one hand, it generates blocks of de‐esterified galacturonic acid residues within the pectin polymer that can be cross‐linked by calcium, thus strengthening the cell‐to‐cell adhesion; on the other hand, the same de‐methyl‐esterified blocks may be more susceptible to degradation by PG. While PG‐mediated polyuronide depolymerization during ripening does not appear to be the primary determinant of tomato fruit softening (Brummell and Harpster, 2001; Giovannoni *et al.*, 1989; Uluisik *et al.*, 2016), experimental data suggest a role for PMEs in determining fruit firmness: silencing of *PMEU1* resulted in faster softening during fruit ripening (Phan *et al.*, 2007), while silencing *PMEU2* results in loss of tissue integrity during fruit senescence (Tieman and Handa, 1994). Additional genes contribute to tomato fruit softening during ripening: *EXP1* and *PL* silencing resulted in a moderate increase in fruit firmness throughout ripening (Brummell *et al.*, 1999; Uluisik *et al.*, 2016; Wang *et al.*, 2019). The expression of genes involved in cell wall remodelling in *LCYb‐*overexpressing fruits is consistent with their phenotypes: *PL, PG* and *EXP1* are down‐regulated, while *PMEU1/2* are up‐regulated compared to *WT*. Taken together, these results indicate that *LCYb* overexpression affects the ripening‐associated pathway leading to pectin solubilization and cell wall disassembly.

*LCYb‐*overexpressing fruits also had significantly higher amounts of cutin monomers and alicyclic wax components relative to *WT*, but unchanged levels of linear, very‐long‐chain wax compounds. Our results suggest a regulatory effect of ABA on cuticle deposition in fruits, consistent with previously published data in tomato (Curvers *et al.*, 2010; Martin *et al.*, 2017) and Arabidopsis (Seo *et al.*, 2011; Zhang *et al.*, 2005) leaves.

### Crosstalk between carotenoids, ABA and ethylene in the control of fruit ripening

Carotenoids, and more specifically 9‐*cis*‐epoxyxanthophylls, synthesized from β‐carotene, are metabolic precursors of ABA (Figure 1A; Giuliano *et al.*, 2003; Qin and Zeevaart, 1999). The increase in ABA levels observed in *LCYb*‐overexpressing fruits is an indirect consequence of the increase in the β‐carotene pool. This is, to some extent, unexpected: the rate‐limiting step for ABA biosynthesis in leaves is believed to be the cleavage of 9‐*cis*‐epoxyxanthophylls by the NCED dioxygenase (Giuliano *et al.*, 2003; Qin and Zeevaart, 1999; Thompson *et al.*, 2000). However, the level of β‐carotene in *WT* tomato fruits is only 1.37‐fold higher than that of ABA and β‐carotene and ABA levels are strictly co‐regulated in *LCYb‐*overexpressing fruits (Tables S16 and S17), suggesting that in tomato fruits, β‐cyclization is rate‐limiting for ABA biosynthesis. Since the β‐cyclization step is itself regulated during ripening (Pecker *et al.*, 1996), this has important implications for the regulatory circuits controlling tomato fruit ripening (see below).

Crosstalk between the ABA and ethylene signalling pathways has been described in Arabidopsis, where a screen for extragenic enhancers or suppressors of ABA‐insensitive *abi1* mutant resulted in alleles of the constitutive ethylene response mutant *ctr1* and ethylene‐insensitive mutant *ein2* (Beaudoin *et al.*, 2000). Blocking of ethylene biosynthesis before véraison in grape, a nonclimacteric fruit, results in inhibition of ABA biosynthesis and of fruit ripening (Sun *et al.*, 2010). This indicates that the crosstalk between ABA and ethylene is probably present in different species and in both climacteric and nonclimacteric fruits. Both positive and negative crosstalk have been reported in tomato fruits between ABA and ethylene, depending on the time and mode of application of the former hormone: exogenous application of ABA during early fruit development induces fruit ethylene biosynthesis and accelerates ripening in a fashion dependent on the *RIN* ripening regulator (Mizrahi *et al.*, 1975; Zhang *et al.*, 2009); exogenous application of ABA or nordihydroguaiaretic acid, an inhibitor of ABA synthesis, respectively, accelerated or delayed fruit ripening, with a simultaneous higher and lower emission in ethylene (Zhang *et al.*, 2009). Furthermore, reduced endogenous ABA levels in fruits of the tomato *hp3* mutant resulted in increased ethylene production (Galpaz *et al.*, 2008). Crosstalk of ABA and ethylene during in tomato fruit ripening has also been observed in fruits with altered expression of transcription factors such as *NAC1* (Meng *et al.*, 2016) and *ZFP2* (Weng *et al.*, 2015).

In *LCYb*‐overexpressing fruits, the increase in ABA levels results in a corresponding decrease in the ethylene peak, a phenotype exactly symmetric to that of the *hp3* mutant (Galpaz *et al.*, 2008). These data support a negative crosstalk between the two hormones. How does this negative crosstalk occur in *LCYb*‐overexpressing fruits? Our data indicate that ABA levels are strongly co‐regulated with transcript levels of several ripening regulators, including *NAC‐NOR* and *RIN* (Table S15 and S16A). *RIN* (Vrebalov *et al.*, 2002) encodes a MADS‐box transcription factor (Ito *et al.*, 2017; Li *et al.*, 2017), and *NAC‐NOR* (Giovannoni *et al.*, 2004) belongs to the NAC domain family of transcription factors, many of which are involved in responses to ABA (Nakashima *et al.*, 2012). Both *NAC‐NOR* and *RIN* are necessary for ethylene synthesis and fruit ripening. NAC‐NOR acts upstream of RIN (Tigchelaar *et al.*, 1973), and RIN binds promoter elements of ripening‐associated genes in a CNR‐dependent fashion (Fujisawa *et al.*, 2013; Martel *et al.*, 2011). Increased *PG* gene expression and increased susceptibility of ripe fruits to *Botrytis cinerea* require *NOR*, but not *RIN* (Cantu *et al.*, 2009; Dellapenna *et al.*, 1987), while induction during ripening of transcripts involved in ethylene biosynthesis is completely abolished in *nor*, but only partially in *rin* fruits (Osorio *et al.*, 2011). ABA accumulation in *rin* fruits is similar to *WT* fruits, while in *nor* fruits it is decreased (McGlasson and Adato, 1976). Two recent studies suggested strong regulatory relationships between *NOR* and ABA: *SlAREB1*, a transcription factor involved in ABA signalling, directly regulates *NOR* expression (Mou *et al.*, 2018) and tomato *de penjar*‐type cultivars, characterized by extended shelf life, displayed a *nor* mutation, increased ABA levels and reduced water loss (Kumar *et al.*, 2018). An interesting hypothesis is that ABA could regulate directly the expression of *NAC‐NOR* and/or *RIN*. A series of ABA response elements such as ABRE, DRE, LTRE, MYB and MYC is localized in the promoter regions of *NOR* and *RIN* (Table S18).

Additional mutants are known to affect β‐carotene biosynthesis in tomato fruits, such as *old gold* and *Beta,* which result respectively in an impairment or an enhancement, of the fruit‐specific *CYC‐b* cyclase (Ronen *et al.*, 2000). An *old gold* allele shows increased fruit firmness, although its fruit ABA content has not been studied (Silletti *et al.*, 2013). *Beta* alleles carry a poorly characterized chromosomal introgression from green‐fruited tomato species, which results in complex vegetative and fruit phenotypes (unpublished data). Additional ABA biosynthesis mutants, such as *flacca* and *sitiens*, exhibit a wilty phenotype and reduced plant and fruit size (Nitsch *et al.*, 2012). These characteristics of the mutants, the fact that the mutations are expressed throughout vegetative growth and fruit ripening, and the hypothesized dual role of ABA in regulating ripening (see below), complicate the interpretation of the mutant phenotypes.

### A model to explain the phenotypic alterations of *LCYb*‐overexpressing fruits

Based on the data gathered, we propose a model to explain the phenotypes observed in *LCYb* fruits. First, β‐carotene levels appear to be the main driver of ABA levels during late ripening, as suggested by the parallel increase of the two metabolites in *LCYb*‐overexpressing fruits. Second, while at the MG stage ABA acts as a trigger for ripening (Zhang *et al.*, 2009), during late ripening it turns into a negative regulator of both ethylene production and fruit ripening (this paper). This dual role of ABA in regulating tomato fruit ripening is supported by the results obtained with RNAi lines where *NCED* silencing is driven by the fruit‐specific *E8* promoter: these lines show decreased ABA and increased ethylene levels during late ripening (Sun *et al.*, 2012). A dual role of ABA in regulating fruit ripening has been also described in peach, where its application at the S3 stage represses ripening, while application at the S4 instead accelerates it (Soto *et al.*, 2013).

**Figure 7:**
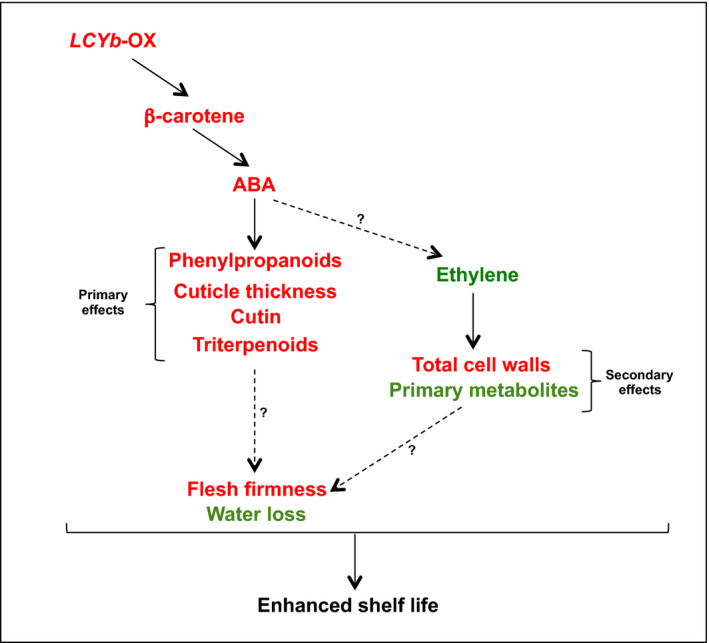
Proposed model for the enhanced shelf life phenotype in *LCYb*‐overexpressing fruits. Variables showing an increase in *LCYb*‐overexpressing fruits are shown in red, and those showing a decrease are shown in green.

In our model (Figure 7), primary effects include those known to be under direct ABA control, such as phenylpropanoid content, cuticle thickness, cutin and triterpenoid composition; secondary effects include instead those not directly associated with ABA, such as cell wall and primary metabolite composition. Since β‐carotene in fruits is mainly synthesized as a result of CYC‐B activity (Ronen *et al.*, 2000), and *CYC‐B* expression is negatively affected by ethylene (Alba *et al.*, 2005), we hypothesize that a negative feedback loop, involving *CYC‐B*, β‐carotene, ABA and ethylene, is active during late fruit ripening, in which ethylene represses *CYC‐B*, lowering the levels of ABA and thus enhancing its own levels and accelerating ripening. In *LCYb*‐overexpressing fruits, lycopene β‐cyclase activity and β‐carotene levels are no more under negative ethylene control, and thus, ABA levels remain high during late ripening, repressing ethylene production and extending shelf life.

Our model is strongly supported by the pharmacological experiment shown in Figure 6: abamine, a known inhibitor of NCED activity, decreases ABA levels in ripe *LCYb*‐overexpressing fruits and simultaneously decreases fruit firmness and water loss and increases ethylene production. This is a strong suggestion that β‐carotene exerts its effects on fruit ripening through its cleavage product, ABA, and not through the alteration of the fruit antioxidant potential, as in the case of the *Del/Ros* tomatoes. Additional studies with inhibitors of ABA synthesis and sensing will shed more light on the role of this hormone in the control of tomato fruit ripening.

## Methods

### Plant material and fruit sampling

The *LCYb* transgenic tomato plants have been previously described (Rosati *et al.*, 2000). Growth of plants was as previously described (Giliberto *et al.*, 2005). Time to breaker was measured by tagging flowers at anthesis. Fruits were harvested at five ripening stages (mature green, MG; breaker, B; and 4, 10 and 15 days after the breaker stage, B+4, B+10 and B+15); at least six fruits from three different plants (three biological replicates) were harvested, cut into small pieces and frozen in liquid nitrogen. Pooled fruits for each biological replicate were stored at −80 °C for a maximum of 6 months before biochemical and transcriptomic analyses.

### Analysis of ABA content

Frozen pericarp tissues at five ripening stages were lyophilized and ground to a fine powder. At least three different pericarp pools (biological replicates) were analysed for each genotype. 200 mg was extracted for each replicate as previously described (Welsch *et al.*, 2008). LC‐HRMS was carried out using a Finnigan Surveyor Plus HPLC System (Thermo Electron), equipped with a 3 μm Hypersil GOLD C18 reverse‐phase column (150 × 4.6 mm; Thermo Electron) as reported before (Ross *et al.*, 2004). Internal standard‐based quantification was carried out using the MS data and the quantification software available in the Xcalibur 2.0 software package (Thermo Fisher Scientific, Bremen, Germany). Retention times and MS^2^ fragmentation patterns were used for identification by using authentic reference standards (trans‐ABA from OlChemIm and (±)‐ABA from Sigma, St. Louis, MO, USA).

### Biochemical and phenotypic assays

Ethylene production was measured on freshly harvested fruits as described (Thompson *et al.*, 1999). At least 10 fruits, with uniform size and pigmentation, were analysed for each line and developmental stage using a Carlo Erba Fractovap 4200 gas chromatograph (Carlo Erba Spa, Milan, Italy) equipped with a flame ionization detector (FID) and 1‐m‐long alumina column (80–100 mesh) at 80 °C. A calibration curve was performed using known concentrations of ethylene. ACO enzymatic activity was assayed according to the protocol from Barry *et al. *(1996). Water loss was measured, for each genotype, on at least 10 fruits from three different plants, kept at constant temperature and relative humidity (22 ± 2 °C, 60 ± 5%). Fruit firmness was evaluated using two different mechanical tests and at least 10 fruits for each line. Pulp firmness was checked on peeled fruits with a 1 kg hand‐held fruit pressure tester (Turoni, Cesena, Italy) equipped with an 8‐mm probe. Pericarp thickness was measured using a calliper. Cuticle isolation was performed on fruits at B+10 as previously described (Saladie *et al.*, 2006). At least four strips/fruit were included in paraffin and fixed in formaldehyde:acetic acid 1:1 (v/v) in 18 volumes of 70% alcohol for 48 h, followed by dehydration in an alcohol series (50%–10%) and a water wash. At least four sections of 10 μm per fruit were cut with a microtome and stained with 0.05% of toluidine blue. Cuticle thickness was determined using light microscope images (40× magnification) and the ImageJ image analysis software (http://rsb.info.nih.gov/ij).

### Cell wall fractionation and composition analysis

Preparation and extraction of total cell wall (TCW) were carried out essentially as described in Orfila *et al. *(2002). Four fractions were extracted sequentially from TCW as reported (Huisman *et al.*, 1998). Monosaccharide composition was determined by high‐performance anion‐exchange chromatography with pulsed amperometric detection (HPAEC‐PAD, Ion Chromatography System, ICS 3000, Dionex, CA) as described (Lionetti *et al.*, 2010). The column was a CarboPac PA20 column 3 × 150 mm (Dionex, CA), equipped with a CarboPac PA20 guard column 3 × 30 mm. Peaks were identified and quantified by comparison to a standard mixture of fucose, rhamnose, arabinose, galactose, glucose, xylose, mannose, galacturonic acid and glucuronic acid (Sigma). All data are expressed as mean ± S.D. Specific pectic epitopes were detected by immunodot assay with LM20 antibody obtained from PlantProbes (UK). Polysaccharide solutions (5 mg/ml) in 0.5% (w/v) ammonium oxalate buffer were spotted as 1 μl drops onto nitrocellulose membrane (Bio‐Rad, Munich, Germany) in a threefold dilution series. Membranes were allowed to air dry at room temperature for 1 h and then blocked with 3% blocking reagent (GE Healthcare, Bucks, UK) in phosphate‐buffered saline (PBS; Bio‐Rad) for 1 h prior to incubation for 1.5 h with LM20. After extensive washes in PBS, membranes were incubated with anti‐rat secondary antibody conjugated to horseradish peroxidase (GE Healthcare) and washed in PBS prior to detection with ECL detection reagent (GE Healthcare). For each analysis, at least three biological replicates were performed.

### Cuticle composition analysis

Cuticular waxes and cutin were analysed as previously described (Yeats *et al.*, 2012). Samples were separated by gas chromatography (5890 II, Hewlett Packard, Avondale, PA; 30 m DB‐1, 0.32 mm i.d., df = 1 μm, J&W Scientific, Folsom, CA) with He carrier gas (1.4 mL/min) and mass spectrometric detection (MS; 5971N, Agilent Technologies, Palo Alto, CA, USA, EI, 70 eV, *m*/*z* 50–800, 1 scan per s.). For analyte quantification, an identical GC system was used, except that a flame ionization detector (FID) was used, which burned H2 (30 mL/min) in air (200 mL/min), and used N2 to shape the flame (20 mL/min). Analytes were quantified against the internal standard by manual integration of peak areas. For each analysis, at least three biological replicates were performed.

### qRT‐PCR and microarray analyses

RNA isolation and real‐time qRT‐PCR conditions were as previously reported (Diretto *et al.*, 2010a). List of primers for each gene is reported in Table S10. Normalization to a housekeeping gene (actin) and to *WT* values was applied to raw data to obtain relative expression levels. For each genotype, at least three biological replicates were performed.

Microarray experiments were carried out using GeneChip^®^ EU‐TOM3 platform (Affymetrix, Buckinghamshire, UK) and an external service provided by IFOM (Fondazione Istituto FIRC di Oncologia Molecolare—Cogentech, Milan, Italy) as previously described (Mori *et al.*, 2012). CEL files were subsequently analysed with RobiNA software (Lohse *et al.*, 2012). Briefly, subsequent steps of quality assessment, data normalization and identification of genes differentially regulated between *WT* and *LCYb*‐overexpressing lines (*LCYb1* and *LCYb3*) fruits for each ripening stage were carried out. The raw data were then normalized using the RMA method. Statistical analysis of pairwise differential gene expression was performed using a linear model‐based approach, applying a 0.05 cut‐off for *P*‐values after a false discovery rate (FDR) correction. Microarray experiments have been deposited to the GEO public repository (https://www.ncbi.nlm.nih.gov/geo) under the accession number GSE108415. For each genotype, at least three biological replicates were performed.

### Primary metabolite analyses

Metabolite analysis of ripe (B+10) tomato peeled pericarp samples (300 mg) by GC‐MS was carried out essentially as described in Lisec *et al. *(2006), with specific modifications for tomato tissues as described in Schauer *et al. *(2006). The GC‐MS system used comprised an AS 2000 autosampler, a GC 8000 gas chromatograph and a Voyager quadrupole mass spectrometer (Thermo Finnigan, Manchester, UK). The mass spectrometer was tuned according to the manufacturer's recommendations using tris‐(perfluorobutyl)‐amine (CF43). Both chromatograms and mass spectra were evaluated using the MASSLAB program (ThermoQuest, Manchester, UK) with reference to libraries of the Golm Metabolite Database (Kopka *et al.*, 2005; Schauer *et al.*, 2005). For each genotype, at least three biological replicates were performed.

### Nonpolar and semi‐polar metabolite analyses

Nonpolar (carotenoids) and semi‐polar (phenylpropanoid) analyses were carried out by liquid chromatography coupled to diode‐array detector and atmospheric pressure chemical ionization–high‐resolution mass spectrometry (LC‐DAD‐APCI‐HRMS) or electrospray ionization (LC‐DAD‐ESI‐HRMS), respectively, operating in positive and negative ion modes, as previously described (D'Esposito *et al.*, 2017; Fasano *et al.*, 2016; Su *et al.*, 2015). Identification of carotenoids was performed as reported previously (Liu *et al.*, 2014). Phenylpropanoid analysis was performed by comparing chromatographic and spectral properties with standards and reference DAD‐HRMS spectra as previously reported (Fernandez‐Moreno *et al.*, 2016; Iijima *et al.*, 2008; Moco *et al.*, 2006). For each analysis, at least three biological replicates were performed.

### Total antioxidant capacity

Fruit total antioxidant capacity was determined as previously described (Enfissi *et al.*, 2010), by estimating the capacity of nonpolar extract to quench the ABTS^+^ radical *via* measuring Abs at 734 nm compared to the one of Trolox. Results were expressed as a TEAC in mm of Trolox per gram of DW. For each line, at least three biological replicates were performed.

### Abamine pharmacological treatment

Abamine treatment of *LCYb*‐overexpressing fruits was carried out as previously described (Brandi *et al.*, 2011; Mou *et al.*, 2016; Su *et al.*, 2015) using, for each fruit, 1 mL of 1 mm abamine, dissolved in dimethylsulphoxide (DMSO). At least 5 MG fruits for each genotype were collected and subjected to treatment with abamine or DMSO (mock). ABA, fruit firmness, ethylene emission and water loss were evaluated as previously described.

### Statistical and bioinformatic analyses

The significance of differences between *WT* and *LCYb*‐overexpressing fruits was evaluated using Student's *t*‐test (*P < 0.05, **P < 0.01). For an easier homogenization and interpretation of the results, all data were normalized on the *WT* values. When the levels of a metabolite or gene expression were ‘not detectable’, an arbitrary value was set, corresponding to 1/10 of the lowest value in the data set. Normalized data were log2‐transformed and visualized on public and *ad hoc* metabolic maps using the MapMan software (Urbanczyk‐Wochniak *et al.*, 2006). For enrichment analysis, Solyc of genes up‐ or down‐regulated was subjected to Gene Ontology Enrichment Analysis (GOEA) using the Plant MetGenMAP tool (http://bioinfo.bti.cornell.edu/tool/GO/GO_enrich.html; Joung *et al.*, 2009). Over‐represented GO terms in each category (biological process, molecular function and cellular component) were determined using the false discovery rate (FDR) statistical method and a p value ≤0.05 (Benjamini and Yekutieli, 2001). Correlation networks were generated by Cytoscape version 2.6.3 (www.cytoscape.org; Cline *et al.*, 2007), as previously described (Aversano *et al.*, 2017; Rambla *et al.*, 2016), and visualized as force‐directed layouts weighted with log_2_ (1−|ρ|) values.

## Author contributions

GG, JG and GD designed the research. GD and AF selected independent transgenic lines. GD and SF performed phenotypic, microarray/qRT‐PCR analyses and carotenoid/phenylpropanoid metabolomic analyses. CF and BM carried out cell wall analyses. NS and ARF performed primary metabolite determinations. ZW, LB and RJ carried out cuticle metabolomics. AJM and JKCR performed cuticle microscopy. All authors analysed data, wrote and agreed the final version of the paper.

## Supporting information


**Figure S1** *AtLCYb *expression in transgenic fruits.
**Figure S2** Time elapsing between anthesis and the breaker stage of fruit maturation in *WT* and *LCYb*‐overexpressors.
**Figure S3** Mapman representations of transcriptional perturbations observed in *LCYb*‐overexpressing fruits at the MG, B and B+10 stages.
**Figure S4** Venn diagram of up‐ and down‐ regulated genes in *LCYb‐*overexpressing fruits at three stages of ripening.
**Figure S5** Correlation network of 1,008 and 790 differentially regulated features using, respectively, ABA (A) and ethylene (B) as central hubs.

**Table S1** Carotenoid composition in *WT* and *LCYb‐*overexpressing fruits at B+10 by LC‐DAD‐MS.
**Table S2** Cutin and wax composition (μg/cm2) of cuticles from WT and *LCYb*‐overexpressing fruits at B+10.
**Table S3** Relative amounts of phenylpropanoids measured in the cuticle and flesh of *WT* and *LCYb*‐overexpressing fruits at B+10.
**Table S4** Relative amounts of primary metabolites in *WT* and *LCYb*‐overexpressing fruits at B+10.
**Table S5** Levels of ABA and ABA catabolites in *WT* and *LCYb‐* overexpressing fruits at 5 ripening stages (MG, B, B+4, B+10 and B+15).
**Table S6** (A) Up‐regulated genes in *LCYb‐*overexpressing MG fruits. (B) Dw‐regulated genes in *LCYb‐*overexpressing MG fruits.
**Table S7** (A) Overrepresented GO terms in all three categories (P: biological process; F: molecular function; C: cellular component) of up‐regulated genes in *LCYb‐*overexpressing fruits at MG. (B) Overrepresented GO terms in all three categories (P: biological process; F: molecular function; C: cellular component) of dw‐regulated genes in *LCYb‐*overexpressing fruits at MG.
**Table S8** (A) Up‐regulated genes in *LCYb‐*overexpressing B fruits. (B) Dw‐regulated genes in *LCYb‐*overexpressing B fruits.
**Table S9** (A) Overrepresented GO terms in all three categories (P: biological process; F: molecular function; C: cellular component) of up‐regulated genes in *LCYb‐*overexpressing fruits at B. (B) Overrepresented GO terms in all three categories (P: biological process; F: molecular function; C: cellular component) of dw‐regulated genes in *LCYb‐*overexpressing fruits at B.
**Table S10** (A) Up‐regulated genes in *LCYb‐*overexpressing B+10 fruits. (B) DW‐regulated genes in *LCYb‐*overexpressing B+10 fruits.
**Table S11** (A) Overrepresented GO terms in all three categories (P: biological process; F: molecular function; C: cellular component) of up‐regulated genes in *LCYb‐*overexpressing fruits at B+10. (B) Overrepresented GO terms in all three categories (P: biological process; F: molecular function; C: cellular component) of dw‐regulated genes in *LCYb‐*overexpressing fruits at B+10.
**Table S12** Common UP‐ and DW‐regulated genes in *LCYb‐*overexpressing fruits in MG/B, B/B+10, MG/B+10 or MG/B/B+10 stages of ripening.
**Table S13** Genes tested by quantitative Real Time PCR, and expression levels in *WT* and *LCYb*‐overexpressing fruits at B+10 stage of ripening.
**Table S14** Correlation matrix of differentially represented genes/metabolites/phenotypes in *LCYb‐*overexpressing B+10 fruits.
**Table S15** Co‐regulation of ripening‐associated genes with ABA and ethylene in *LCYb*‐overexpressing fruits.
**Table S16** Pearson correlation coefficients (ρ≥|0.90|) between differentially regulated genes/metabolites/phenotypes and ABA (A), or Ethylene (B) in *LCYb‐*overexpressing B+10 fruits.
**Table S17** Lycopene, β‐carotene and ABA‐ molar concentrations in *WT* and *LCYb‐*overexpressing B+10 tomato fruits.
**Table S18** ABA‐ and stress‐ response elements sites found in the promoters of ethylene‐regulators.
