# Supplementary material for: Manipulation of β‐carotene levels in tomato fruits results in increased ABA content and extended shelf life
Source: Plant Biotechnol J. 2019 Dec 24;18(5):1185–99. doi: 10.1111/pbi.13283 (PMC7152610; doi:10.1111/pbi.13283)
Supplement: Supplementary file 1 — Figure S1 AtLCYb expression in transgenic fruits. Figure S2 Time elapsing between anthesis and the breaker stage of fruit maturation in WT and LCYb‐overexpressors. Figure S3 Mapman representations of transcriptional perturbations observed in LCYb‐overexpressing fruits at the MG, B and B+10 stages. Figure S4 Venn diagram of up‐ and down‐ regulated genes in LCYb‐overexpressing fruits at three stages of ripening. Figure S5 Correlation network of 1,008 and 790 differentially regulated features using, respectively, ABA (A) and ethylene (B) as central hubs. [file PBI-18-1185-s002.pptx]

## Slide 1
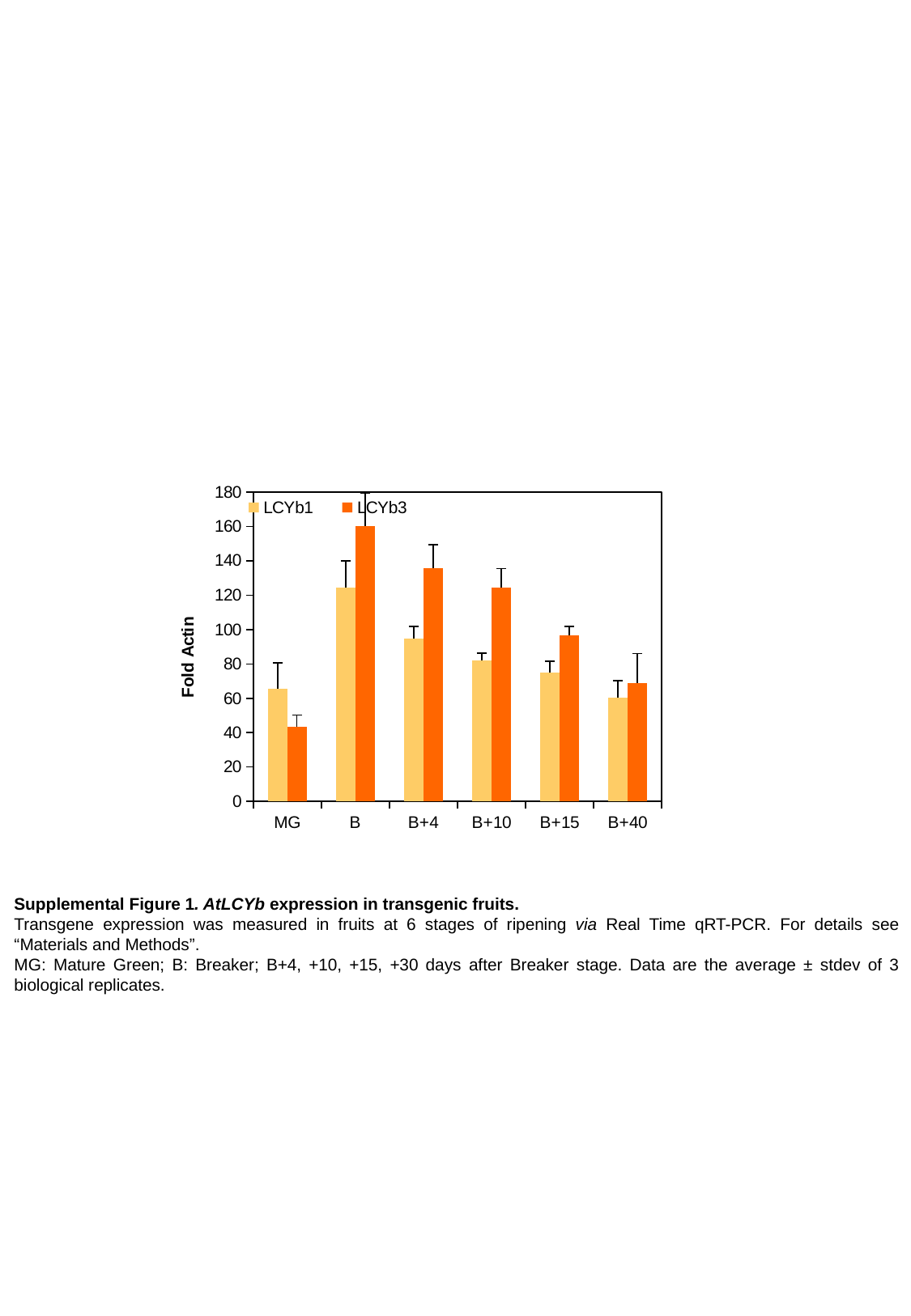

### Chart
| Category | LCYb1 | LCYb3 |
|---|---|---|
| MG | 65.7732398688294 | 43.17784674 |
| B | 124.5667088857448 | 160.25783647 |
| B+4 | 94.81822100846665 | 135.8226802 |
| B+10 | 81.79334281884552 | 124.3652784 |
| B+15 | 74.81730219999997 | 96.48295299999998 |
| B+40 | 60.15167374563 | 68.8815167483 |Supplemental Figure 1. AtLCYb expression in transgenic fruits.
Transgene expression was measured in fruits at 6 stages of ripening via Real Time qRT-PCR. For details see “Materials and Methods”.
MG: Mature Green; B: Breaker; B+4, +10, +15, +30 days after Breaker stage. Data are the average ± stdev of 3 biological replicates.

## Slide 2
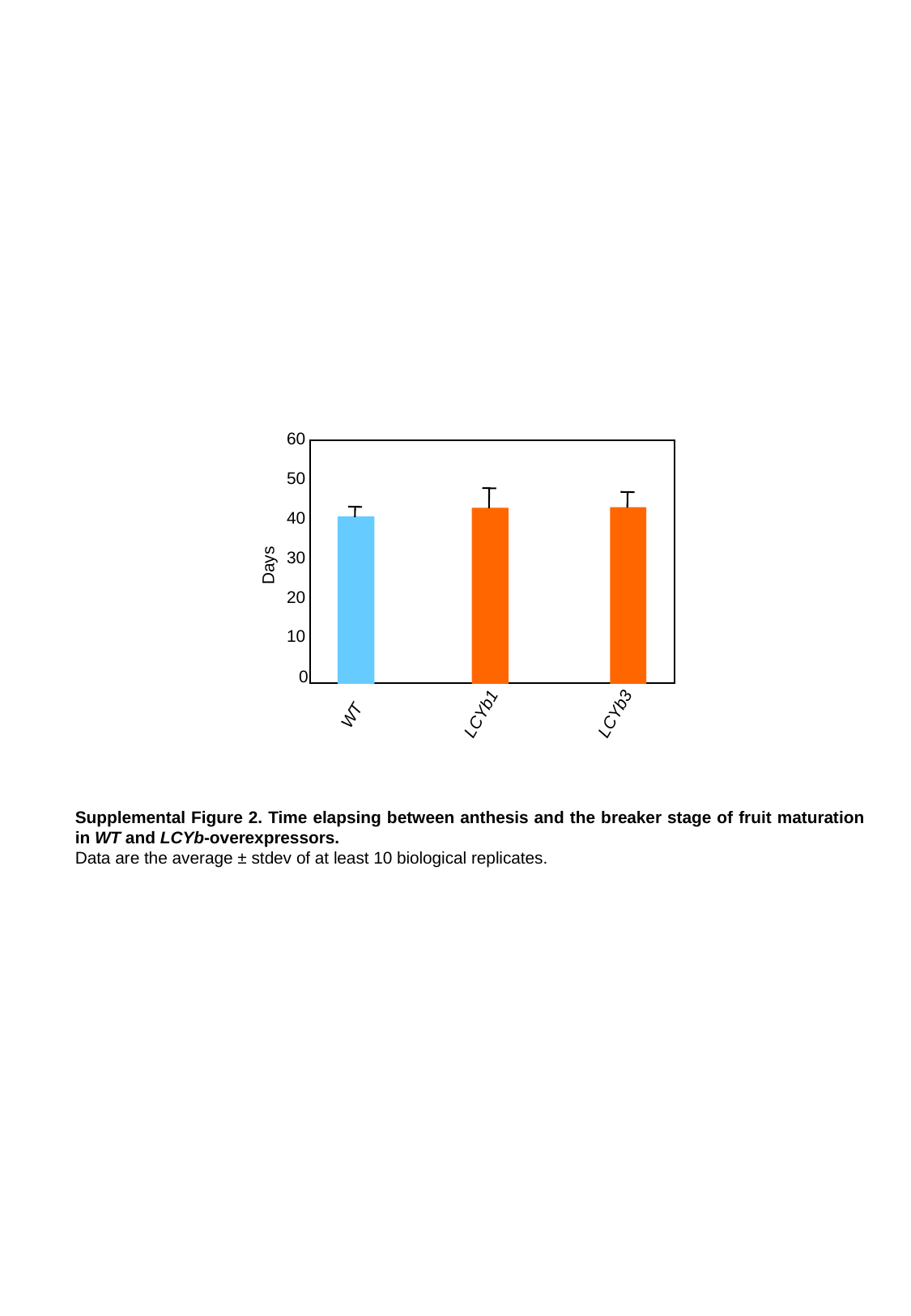

60
50
LCYb1
LCYb3
WT
40
30
Days
20
10
0
Supplemental Figure 2. Time elapsing between anthesis and the breaker stage of fruit maturation in WT and LCYb-overexpressors.
Data are the average ± stdev of at least 10 biological replicates.

## Slide 3
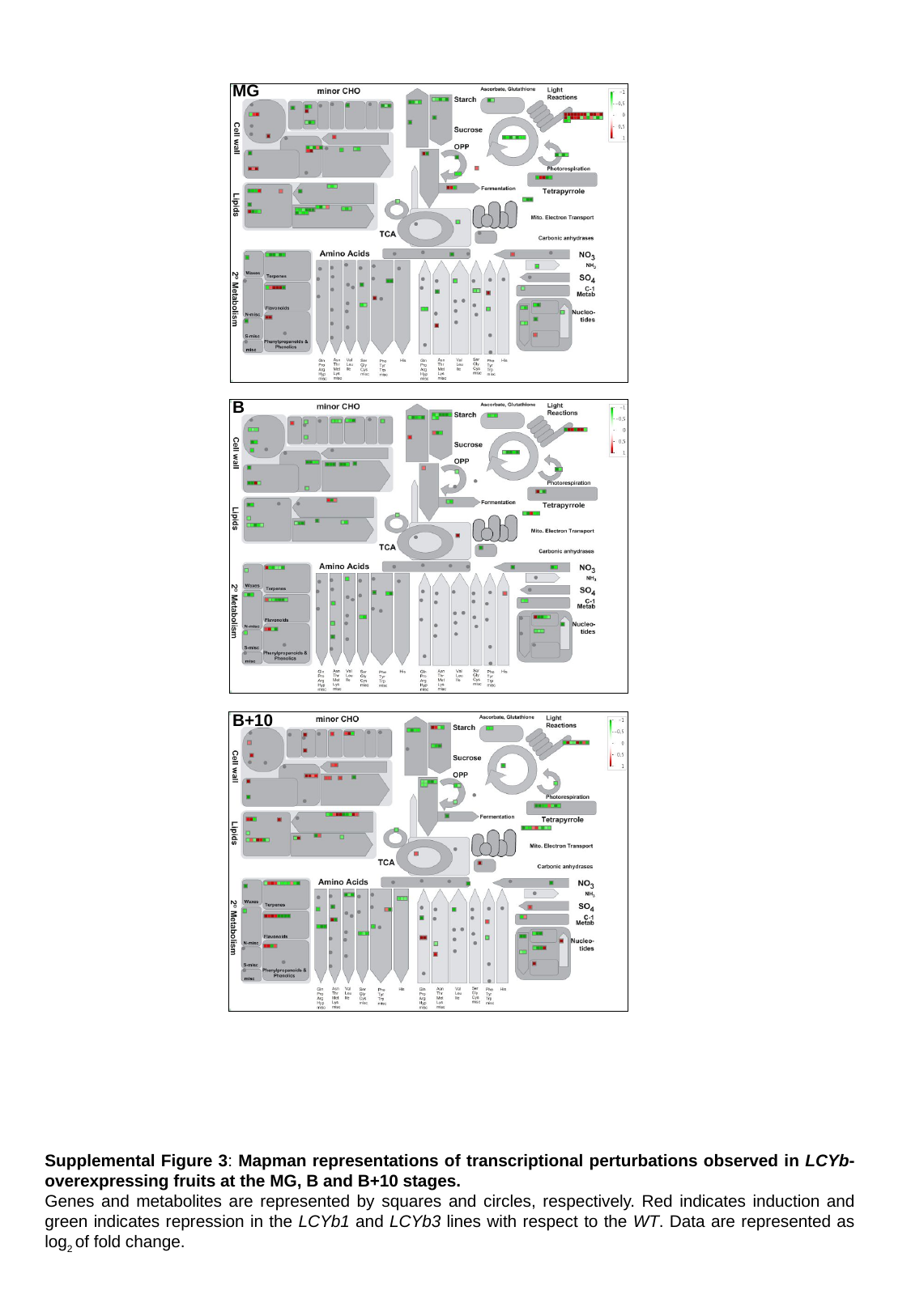

MG
B
B+10
Supplemental Figure 3: Mapman representations of transcriptional perturbations observed in LCYb-overexpressing fruits at the MG, B and B+10 stages.
Genes and metabolites are represented by squares and circles, respectively. Red indicates induction and green indicates repression in the LCYb1 and LCYb3 lines with respect to the WT. Data are represented as log2 of fold change.

## Slide 4
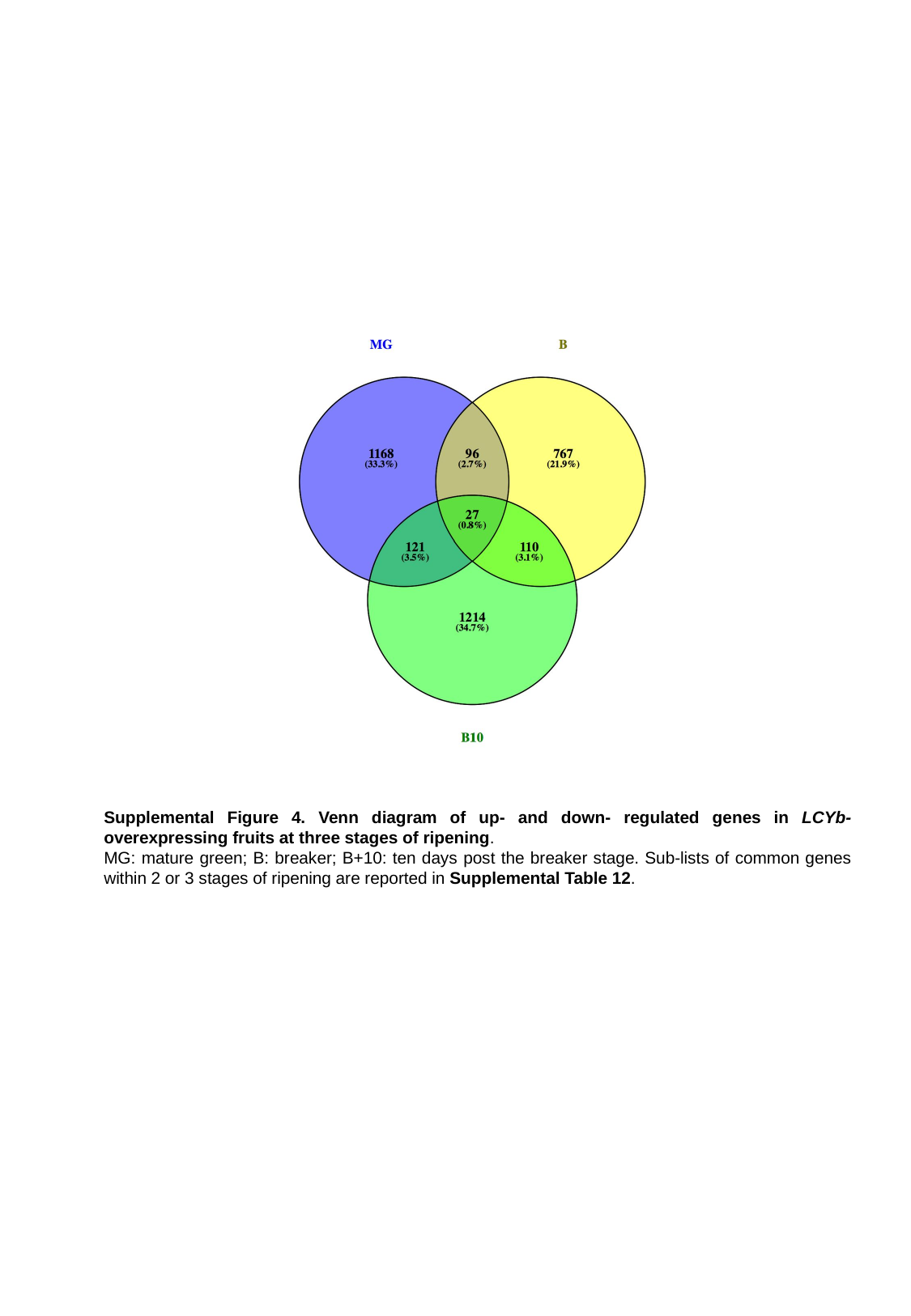

Supplemental Figure 4. Venn diagram of up- and down- regulated genes in LCYb-overexpressing fruits at three stages of ripening.
MG: mature green; B: breaker; B+10: ten days post the breaker stage. Sub-lists of common genes within 2 or 3 stages of ripening are reported in Supplemental Table 12.

## Slide 5
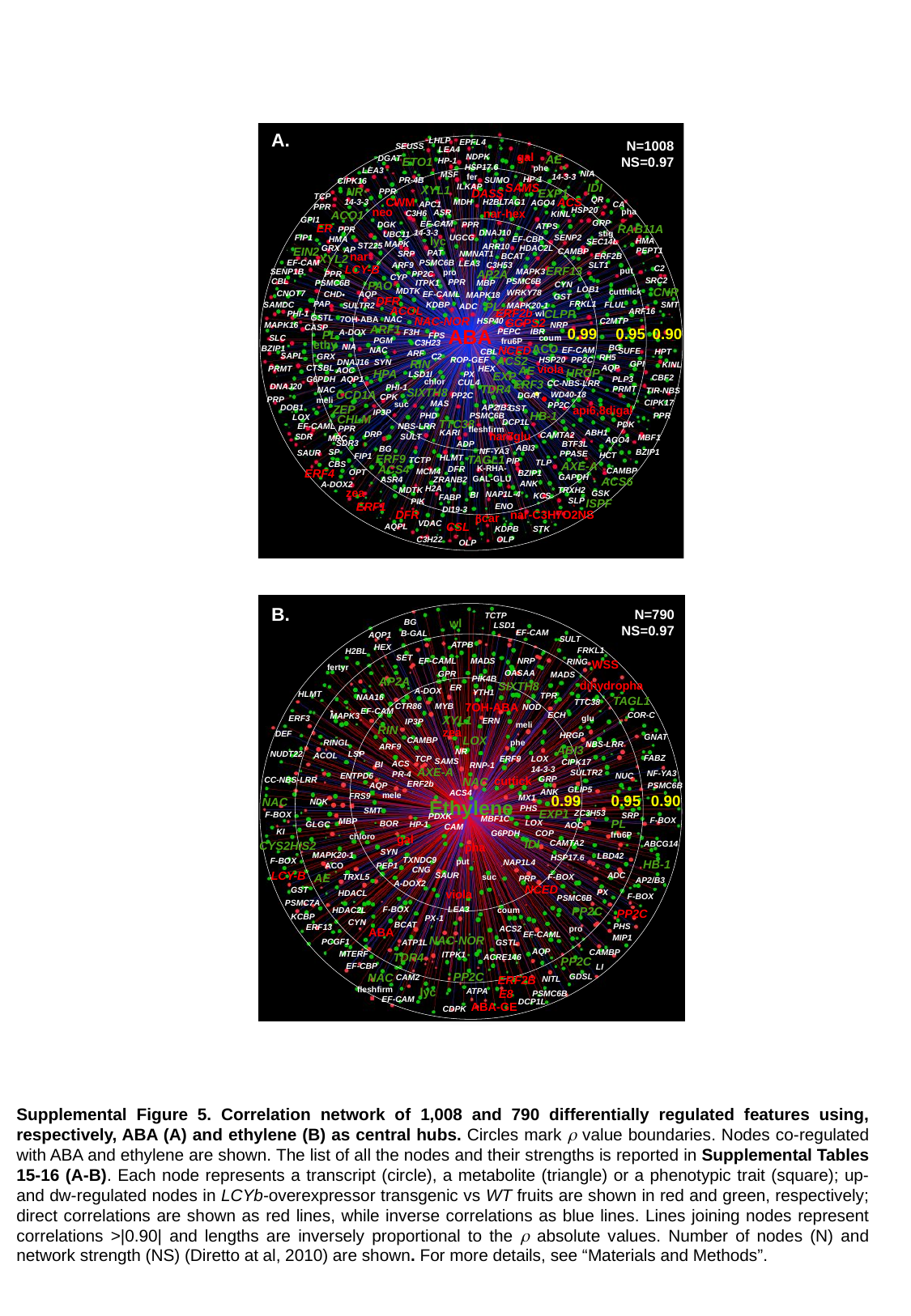

A.
LHLP
EPFL4
N=1008
NS=0.97
SEUSS
LEA4
gal
NDPK
AE
DGAT
ETO1
HP-1
HSP17.6
phe
LEA3
NIA
MSF
fer
14-3-3
HP-1
PR-4B
SUMO
CIPK16
SAMS
IDI
ILKAP
XYL1
NR
DASS
PPR
EXP1
TCP
QR
ACS
CWM
H2BL
14-3-3
MDH
TAG1
AGO4
CA
APC1
PPR
HSP20
neo
nar-hex
pha
ASR
C3H6
ACO1
KINL
GPI1
GRP
EF-CAM
DGK
PPR
ER
ATPS
RAB11A
PPR
DNAJ10
14-3-3
stig
UBC11
UGCG
SENP2
FIP1
EF-CBP
HMA
lyc
HMA
SEC14L
MAPK
ST225
ARR10
HDAC2L
GRX
EIN2
AP
PEPT1
CAMBP
PAT
SRP
NMNAT1
nar
BCAT
ERF2B
XYL2
EF-CAM
PSMC6B
LEA3
ARF9
C3H53
SLT1
LCY-B
C2
ERF13
put
SENP1B
MAPK3
pro
AP2A
PP2C
PPR
CYP
SRC2
PSMC6B
CBL
PPR
ITPK1
PSMC6B
MBP
PAO
CYN
LOB1
CNR
MDTK
cutthick
WRKY78
CNOT7
EF-CAML
CHD
AQP
MAPK18
GST
DFR
FRKL1
PAP
SAMDC
KDBP
PL
FLUL
SMT
MAPK20-1
SULTR2
ADC
ACOL
ERF2b
ARF16
CLPP
wl
PHI-1
GSTL
NAC-NOR
7OH-ABA
NAC
GGPS2
C2MTP
HSP40
MAPK16
NRP
CASP
ARF1
ABA
0.99
0.95
0.90
PEPC
IBR
A-DOX
F3H
PL
FPS
SLC
coum
PGM
fru6P
ethy
C3H23
NIA
ACO
BG
NCED
BZIP1
EF-CAM
NAC
SUFE
CBL
HPT
ARF
SAPL
C2
GRX
RH5
ACS2
HSP20
PP2C
ROP-GEF
RIN
SYN
DNAJ16
GPI
KINL
viola
AQP
CTSBL
HEX
AE
PRMT
AOC
HRGP
HPA
EXP
LSD1l
PX
CBF2
AQP1
PLP3
G6PDH
chlor
ERF3
CUL4
CC-NBS-LRR
DNAJ20
TDR4
PHI-1
PRMT
NAC
TIR-NBS
SIXTH8
CCD1A
WD40-18
DGAT
PP2C
CPK
PRP
meli
CIPK17
MAS
suc
PP2C
ZEP
DOB1
AP2/B3
GST
api6,8digal
IP3P
HB-1
PSMC6B
PHD
PPR
CHLM
LOX
TTC38
DCP1L
PDK
NBS-LRR
EF-CAML
PPR
fleshfirm
ABH1
KARI
nar7glu
DRP
CAMTA2
SULT
SDR
MBF1
MRC
AGO4
SDR3
BTF3L
ADP
ABI3
BG
NF-YA3
SP
BZIP1
SAUR
PPASE
HCT
FIP1
ERF9
TAGL1
HLMT
TCTP
PIP
TLP
AXE-A
CBS
ACS4
K-RHA-
GAL-GLU
DFR
CAMBP
ERF4
MCM4
OPT
BZIP1
GAPDH
ACS6
ASR4
ZRANB2
ANK
A-DOX2
H2A
MDTK
TRXH2
zea
GSK
NAP1L-4
BI
KCS
FABP
SLP
ISPF
PIK
ERF1
ENO
DI19-3
DFR
nar-C3H7O2NS
bcar
VDAC
CSL
AQPL
KDPB
STK
C3H22
OLP
OLP
B.
N=790
NS=0.97
TCTP
wl
BG
LSD1
EF-CAM
B-GAL
AQP1
SULT
ATPB
HEX
FRKL1
H2BL
SET
NRP
EF-CAML
MADS
RING
WSS
fertyr
OASAA
GPR
MADS
PIK4B
AP2A
dihydropha
SIXTH8
ER
A-DOX
YTH1
HLMT
TPR
NAA16
TAGL1
TTC38
7OH-ABA
CTR86
MYB
NOD
EF-CAM
COR-C
ECH
MAPK3
ERF3
XYL1
glu
ERN
IP3P
meli
RIN
zea
DEF
HRGP
GNAT
LOX
CAMBP
RINGL
phe
NBS-LRR
ARF9
ABI3
NR
NUDT22
LSP
ACOL
FABZ
TCP
ERF9
LOX
SAMS
CIPK17
ACS
BI
RNP-1
14-3-3
AXE-A
SULTR2
NF-YA3
PR-4
NUC
ENTPD6
GRP
cuttick
NAC
CC-NBS-LRR
ERF2b
AQP
PSMC6B
GLIP5
ANK
ACS4
mele
FRS9
0.99
0.95
0.90
MX1
NAC
Ethylene
NDK
PHS
SMT
EXP1
ZC3H53
F-BOX
SRP
PDXK
MBF1C
F-BOX
MBP
PL
LOX
BOR
HP-1
GLGC
AOC
CAM
KI
COP
G6PDH
fru6P
chloro
gal
IDI
CAMTA2
CYS2HIS2
ABCG14
pha
SYN
MAPK20-1
LBD42
HSP17.6
TXNDC9
F-BOX
put
NAP1L4
HB-1
PEP1
ACO
CNG
LCY-B
SAUR
ADC
AE
suc
TRXL5
F-BOX
PRP
AP2/B3
A-DOX2
NCED
GST
viola
PX
HDACL
F-BOX
PSMC6B
PSMC7A
LEA3
PP2C
F-BOX
coum
HDAC2L
PP2C
KCBP
PX-1
CYN
BCAT
PHS
ERF13
pro
ACS2
ABA
EF-CAML
NAC-NOR
MIP1
PCGF1
ATP1L
GSTL
AQP
CAMBP
MTERF
ITPK1
TDR4
ACRE146
PP2C
EF-CBP
LI
PP2C
NAC
GDSL
CAM2
ERF2B
NITL
fleshfirm
lyc
ATPA
E8
PSMC6B
EF-CAM
DCP1L
ABA-GE
CDPK
Supplemental Figure 5. Correlation network of 1,008 and 790 differentially regulated features using, respectively, ABA (A) and ethylene (B) as central hubs. Circles mark r value boundaries. Nodes co-regulated with ABA and ethylene are shown. The list of all the nodes and their strengths is reported in Supplemental Tables 15-16 (A-B). Each node represents a transcript (circle), a metabolite (triangle) or a phenotypic trait (square); up- and dw-regulated nodes in LCYb-overexpressor transgenic vs WT fruits are shown in red and green, respectively; direct correlations are shown as red lines, while inverse correlations as blue lines. Lines joining nodes represent correlations >|0.90| and lengths are inversely proportional to the r absolute values. Number of nodes (N) and network strength (NS) (Diretto at al, 2010) are shown. For more details, see “Materials and Methods”.
